# Transmission and microevolution of methicillin-resistant *Staphylococcus aureus* ST88 strain among patients, healthcare workers, and household contacts at a trauma and orthopedic ward

**DOI:** 10.3389/fpubh.2022.1053785

**Published:** 2023-01-09

**Authors:** Long Sun, Hemu Zhuang, Lingfang Di, Xia Ling, Yiping Yin, Zhengan Wang, Mengzhen Chen, Shengnan Jiang, Yiyi Chen, Feiteng Zhu, Haiping Wang, Shujuan Ji, Lu Sun, Dandan Wu, Yunsong Yu, Yan Chen

**Affiliations:** ^1^Department of Clinical Laboratory, Hangzhou Women's Hospital (Hangzhou Maternity and Child Health Care Hospital), Zhejiang, China; ^2^Department of Infectious Diseases, Sir Run Run Shaw Hospital, Zhejiang University School of Medicine, Hangzhou, China; ^3^Key Laboratory of Microbial Technology and Bioinformatics of Zhejiang Province, Hangzhou, Zhejiang, China; ^4^Regional Medical Center for National Institute of Respiratory Diseases, Sir Run Run Shaw Hospital, Zhejiang University School of Medicine, Hangzhou, China; ^5^Department of Clinical Laboratory, Tongxiang First People's Hospital, Tongxiang, Zhejiang, China; ^6^Blood Center of Zhejiang Province, Hangzhou, Zhejiang, China; ^7^Hospital Infection Control Office, Hospital of Zhejiang People's Armed Police, Zhejiang, China; ^8^Department of Infectious Diseases, Second Affiliated Hospital, Zhejiang University School of Medicine, Hangzhou, Zhejiang, China

**Keywords:** whole-genome sequencing, methicillin-resistant *Staphylococcus aureus*, surgical site infection, hospital, colonization

## Abstract

**Background:**

Surgical sites infections (SSIs) caused by Methicillin-resistant *Staphylococcus aureus* (MRSA) constitute a major clinical problem. Understanding the transmission mode of MRSA is important for its prevention and control.

**Aim:**

We investigated the transmission mode of a MRSA outbreak in a trauma and orthopedic hospital ward.

**Methods:**

Clinical data were collected from patients (*n* = 9) with MRSA infection in a trauma and orthopedic ward from January 1, 2015 to December 31, 2019. The wards (*n* = 18), patients (*n* = 48), medical staff (*n* = 23), and their households (*n* = 5) were screened for MRSA. The transmission mode of MRSA isolates was investigated using next-generation sequencing and phylogenetic analyses. The resistance genes, plasmids, and single-nucleotide variants of the isolates were analyzed to evaluate microevolution of MRSA isolates causing SSIs. The MRSA colonization-positive doctor was asked to suspend his medical activities to stop MRSA spread.

**Findings:**

Nine MRSA infected patients were investigated, of which three patients were diagnosed with SSI and had prolonged hospitalization due to the persistent MRSA infection. After screening, MRSA isolates were not detected in environmental samples. The surgeon in charge of the patients with SSI caused by MRSA and his son were positive for MRSA colonization. The MRSA from the son was closely related to the isolates detected in MRSA-induced SSIs patients with 8–9 single-nucleotide variants, while ST88-MRSA isolates with three different *spa* types were detected in the surgeon's nasal cavity. Comparative genomic analysis showed that ST88-MRSA isolates acquired mutations in genes related to cell wall synthesis, colonization, metabolism, and virulence during their transmission. Suspending the medical activity of this surgeon interrupted the spread of MRSA infection in this ward.

**Conclusion:**

Community-associated MRSA clones can invade hospitals and cause severe postoperative nosocomial infections. Further MRSA surveillance in the households of health workers may prevent the transition of MRSA from colonization to infection.

## 1. Introduction

Surgical site infection (SSI) after orthopedic surgery accounts for 14%−16% of all nosocomial infections and prolongs hospital stays by a median of 14 days per patient, increases hospitalization expenses by more than three-fold, and approximately doubles rehospitalization rates (1). Given the increasing infection rate of methicillin-resistant *Staphylococcus aureus* (MRSA), *S. aureus* is among the most important pathogens in SSIs (2). *Staphylococcus aureus* is a common pathogen; however, upon acquiring the staphylococcal cassette chromosome *mec* element, *S. aureus* can become resistant to almost all β-lactam antibiotics, giving rise to MRSA (3). Recently, because of increases in hypervirulent and low fitness-cost strains, community-acquired MRSA (CA-MRSA) clones are causing infections in hospitalized patients (4). Unlike hospital-acquired MRSA, CA-MRSA often causes infections in young adults (5). Acute CA-MRSA infection can develop rapidly and cause the death of the patient (6). Depending on the type of surgery performed and carrier status of individuals, the incidence of MRSA SSIs varies from 1 to 33% (7). Blocking the transmission of pathogens can effectively reduce the infection rate of hospitalized patients, the number of days in the hospital, and medical expenses.

Understanding the mechanism by which pathogens spread is a key factor in blocking their dissemination; however, the pattern of CA-MRSA infiltration into hospitals is poorly understood. Sequencing technology can be used to accurately identify and explore the characteristics of spreading pathogens (8, 9). Using genomic surveillance, Thiede et al. (10) revealed that the most prevalent CA-MRSA lineage, USA300, was circulating across both community and healthcare settings.

This study was conducted to investigate an *S. aureus* infection outbreak that occurred in a trauma and orthopedic ward of a secondary hospital. Medical records were reviewed, and the patients, medical staff, and households of the staff were screened for MRSA. Phylogenetic analysis was performed using whole-genome sequencing.

## 2. Methods

### 2.1. Clinical cases and isolates

This study was conducted in a trauma and orthopedic ward with 45 beds in a hospital with 886 admissions per year. A total of nine cases of MRSA infection from January 1, 2015, to December 31, 2019, were included in this study. MRSA isolates were identified using a microTyper MS analyzer (Skyray, Jiangsu, China). Patient information was obtained from electronic medical records. All collected isolates were preserved at −80°C. The local ethics committee of Sir Run Run Shaw Hospital reviewed and approved this study (number 20190821-9). The patients/participants provided their consent to participate in this study.

### 2.2. MRSA screening

To trace the origin of the outbreak of MRSA infections in patients after bone and joint surgery, we conducted environmental MRSA screening in this trauma and orthopedic ward in May 2017. Several sample types were collected for MRSA screening. Air samples were collected from the ward using the sedimentation method. Opened Columbia blood agar plates were evenly placed in the east, south, west, north, and middle of the ward to allow microorganisms to settle as previously described (11). Samples from object surfaces were collected using saline-moistened cotton swabs, which were inoculated on CHROMagar Staph aureus plates (CHROMagar, Paris, France). Environmental samples were collected from bed sheets, bed rails, curtains, quilt covers, tabletops, toilet seats, and medical devices such as stethoscopes that were present in the ward. MRSA colonization screening were also conducted for the patients, healthcare staff and households of healthcare staff. Swab samples were taken from their nasal cavities and hands. For MRSA positive individual, the groins and armpits were also screened.

### 2.3. Antibiotic susceptibility testing

Antibiotic susceptibility testing of all isolates was performed using a BD Phoenix™-100 Automated Microbiology System (BD Biosciences, Franklin Lakes, NJ, USA). For the sequenced isolates, a drug susceptibility test was performed according to Clinical and Laboratory Standards Institute guidelines (12).

### 2.4. Genome sequencing

A primary MRSA isolate from each patient with MRSA infection was selected for sequencing. Since the MRSA isolates isolated from a patient in 2015 was not stocked, we sequenced MRSA isolates from eight patients as listed in Table 1. From the staff and the households, at least one representative isolate from each site was selected for sequencing (Table 1).

**Table 1:** Information on MRSA sequencing strains isolated in this study.

**Source**	**Diagnosis**	**Isolates ID**	**Specimen type**	**Isolation date**	**ST**
Patient [1]	SSI	P1T1	Secretions	2016-02-25	88
		P1T2	Secretions	2016-06-14	88
Patient [2]	SSI	P2	Secretions	2016-05-16	88
Patient [3]	SSTI	P3	Secretions	2016-10-10	59
Patient [4]	Burn	P4	Secretions	2016-10-19	88
		P4T6^a^	Secretions	2016-10-19	7
Patient [5]	SSI	P5T1	Secretions	2017-03-24	88
		P5T2^b^	Secretions	2017-05-10	88
Patient [6]	SSI	P6	Secretions	2017-04-02	398
Patient [7]	SSTI	P7	Secretions	2017-07-31	630
Patient [8]	SSTI	P8	Secretions	2017-11-16	338
Doctor	Colonization	DN1	Nasal swab	2017-05-11	88
		DN2	Nasal swab	2017-05-11	88
		DN3	Nasal swab	2017-05-11	88
		DN4	Nasal swab	2017-05-11	88
		DA	Skin swab	2017-05-12	88
		DG	Skin swab	2017-05-12	88
Son of the doctor	Colonization	SN1	Nasal swab	2017-05-17	88

a Simultaneous isolation of MRSA and MSSA from patient 4. The relationship between these two strains was evaluated by sequencing. P4T6 was MSSA. SSTI, skin and soft tissue infection.

b Two MRSA isolates isolated from patient 5 were sequenced to explore the potential strain evolution.

Total DNA was extracted from the MRSA isolates using a QIAamp DNA Mini Kit (Qiagen, Hilden, Germany). The extracted DNA was sequenced on an Illumina HiSeq Xten platform (San Diego, CA, USA) using the 2 × 150 base pair paired-end mode. The derived short reads were assembled into a FASTA file using the Shovill pipeline (version 4.4.5, https://github.com/tseemann/shovill). The minimum length and depth of Shovill were set to 200 and 10, respectively.

### 2.5. Molecular typing

The assemblies of sequencing data were entered into SeqSphere+ software (version 4.1.9; Ridom GmbH, Münster, Germany) for multilocus sequence typing, *spa*, and core genome multilocus sequence typing analyses using default parameters. The minimum spanning trees of these MRSA isolates were constructed based on differences in 1,788 alleles.

### 2.6. Plasmid and resistance gene detection

Plasmids and resistance genes were detected using the ABRIcate pipeline (version 1.0.0, https://github.com/tseemann/abricate). PlasmidFinder (13) and NCBI AMRFinderPlus (14) were used for plasmid and resistance gene screening, respectively. The thresholds for coverage and identity were 80 and 75%, respectively. Using the genome of an ST88-MRSA strain previously isolated from an SSI (15), the plasmid map was constructed by placing the FASTA file in BRIG (version 0.95).

### 2.7. Plasmid stability test

To verify that the plasmids carrying the erythromycin resistance gene (pSR02) in these strains are easily lost, passaging experiments were performed. Isolates P1, DN4, and SN1 were passaged in tryptic soy broth at 37 °C. The bacterial solution was collected every other day, cultivated on a tryptic soy agar (TSA) plate to select single clones, and simultaneously inoculated onto TSA and TSA containing 10 μg/ml erythromycin plates. The day on which clones grew on the TSA plates but not on the TSA plates containing erythromycin was recorded.

### 2.8. Phylogeny analysis

The FASTA file was annotated using the Prokka pipeline (version 1.14.6) (16). The annotated general feature format file was entered into the Panaroo pipeline (version 1.2.7) (17) in strict mode to generate a core-genome alignment file. The alignment file was placed in the IQ-TREE pipeline (version 2.1.2) (18) to generate a maximum-likelihood tree. The model for the tree was selected using ModelFinder (19). The number of bootstrap replicates was selected as 1,000 as recommended by the IQ-TREE Operation manual using UFBoot (20). Single-nucleotide variants (SNVs) were calculated using the Snippy pipeline (version 4.4.5, https://github.com/tseemann/snippy). A total of 2,516 core genes in the MRSA isolates were included in the phylogenetic analysis and SNV counting.

### 2.9. Screening of amino acid mutation sites

The P1T1 strain was the earliest MRSA strain isolated the index patient. Using this annotated strain as a reference, specific amino acid mutation sites were detected in our ST88-MRSA isolates using the Snippy pipeline (version 4.4.5, https://github.com/tseemann/snippy).

## 3. Results

### 3.1. MRSA outbreak in the trauma and orthopedic hospital ward

From February, 2016, to November, 2017, we observed that the MRSA infection rate was significantly higher than those recorded in 2014–2015 in this ward (Figure 2B), indicating a potential outbreak of MRSA, with eight MRSA-infected patients detected in 2016–2017, including four with SSI, three with skin and soft tissue infection, and one with burn wound infection (Table 1). A review of the medical data revealed that the three patients with SSI were treated by the same doctor (doctor D), indicating postoperative nosocomial infection.

**Figure 1:**
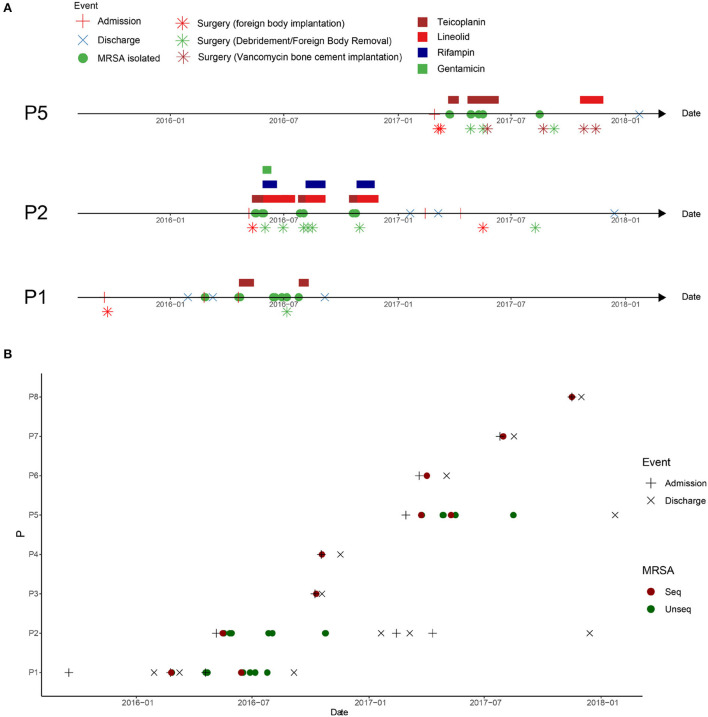
Clinical events in patients with methicillin-resistant *Staphylococcus aureus* (MRSA) infection in the orthopedic surgery ward. **(A)** Clinical events in patients with MRSA infection at the surgical site after osteoarthritis surgery. P1: patient 1, P2: patient 2, and P5: patient 5. **(B)** Timeline of MRSA isolation from MRSA-infected patients in this ward between admission of P1 and discharge of P5. The dotted line represents the screening time of the medical staff in the ward.

Patient 1 (P1), a 21-year-old man, was admitted to the hospital with a right knee injury on September 17, 2015. On day six of admission, the patient underwent arthroscopic anterior cruciate ligament reconstruction surgery by doctor D. The patient was discharged from the hospital on January 29, 2016. One month after discharge, his right knee became swollen and ulcerated. On February 24, 2016, the patient was re-hospitalized for treatment. MRSA was isolated from wound secretions at that time, but the patient was not treated with anti-MRSA therapy and was discharged after 13 days. Because of rupture of the front of the right knee joint, the patient was admitted to the hospital for the third time on April 19, 2016. The patient continuously tested positive for MRSA during hospitalization and was administered teicoplanin. The patient subsequently underwent right knee surgery, during which the steel plates and screws were removed, and debridement was performed. However, MRSA was still continuously recovered in the secretions of the knee joint after surgery (Figure 1A).

Patient 2 (P2), a 20-year-old man, was admitted to the hospital with an ankle fracture on May 6, 2016. Six days later, he underwent surgery on his right ankle performed by doctor D. After surgery, MRSA isolates were continuously isolated from the secretions at the surgical incision, and linezolid was used for treatment (Figure 1A, P2).

Patient 5 (P5), a 21-year-old man, was admitted to the hospital with ankle and fibular fractures on February 28, 2017. He underwent surgery for right ankle skeletal traction by doctor D on March 6, 2017. Four days later, the patient underwent right ankle fracture resection and internal fixation. Two weeks later, secretions from the surgical incision tested positive for MRSA. The doctors performed debridement and applied vancomycin bone cement (Figure 1A, P5).

To investigate the spread of MRSA isolates in the hospital, the medical records of all MRSA-infected patients in this ward during the MRSA outbreak period were reviewed. Eight cases of MRSA infection were reported from February, 2016 to May, 2017. The hospitalization period of these patients overlapped (Figure 1B).

### 3.2. MRSA screening statistics

To clarify the transmission mode of MRSA in the ward, medical staff (*n* = 23), households (*n* = 5) of hospital staff harboring MRSA, patients (*n* = 48) who stayed in the same ward as those with MRSA infection during the study period, and the ward (*n* = 18) environment were screened for MRSA. In total, 194 environmental samples and 156 samples from patients, hospital staffs, and households of hospital staffs were collected. No MRSA was isolated from the environmental samples. In addition, no MRSA isolates were found in samples obtained from the hands or nasal cavity of 48 patients (Table 2).

**Table 2:** MRSA screening sampling statistics.

**Sample object**	**Number of specimens**	**Source**	**Number of MRSA cases**
Rooms (*n* = 18)	194	Air (*n* = 90)	0
		Object surface^a^ (*n* = 104)	0
Patients (*n* = 48)	96	Hand (*n* = 48)	0
		Nasal cavity (*n* = 48)	0
Hospital staff (*n* = 23)	48	Hand (*n* = 23)	0
		Nasal cavity (*n* = 23)	1 (Doctor D)
		Groin (*n* = 1)	1 (Doctor D)
		Armpit (*n* = 1)	1 (Doctor D)
Households of hospital staff (*n* = 5)	10	Hand (*n* = 5)	0
		Nasal cavity (*n* = 5)	1 (Son of Doctor D)

^a^Object surface including bed sheets, bed rails, curtains, quilt covers, tabletops, toilet seats, and medical devices such as stethoscopes.

Among the 23 medical staffs who worked in this ward, MRSA was isolated only from the nasal cavity of Doctor D, who was the doctor in charge of patients P1, P2, and P5. Further screening revealed that this doctor also had MRSA colonization in the armpit and groin.

To further explore the transmission of MRSA, the households of Doctor D, including his wife and son, were screened for MRSA colonization. One MRSA isolate (SN1) was obtained from the nasal cavity of his 5-year-old son (Table 2). This result indicated that these MRSA strains may have spread among the patients, doctor D, and his son.

### 3.3. Molecular typing and phylogenetic analysis

Among the 18 MRSA isolates recovered from the patients (*n* = 11), Doctor D (*n* = 6), and the household of Doctor D (*n* = 1), six STs were identified, including ST88, ST59, ST630, ST338, ST398, and ST7. Based on the differences in the core gene alleles, the main epidemic MRSA clone in this department was ST88, which was isolated from P1, P2, P4, P5, Doctor D, and SN1 (Supplementary Figure 1).

**Figure 2:**
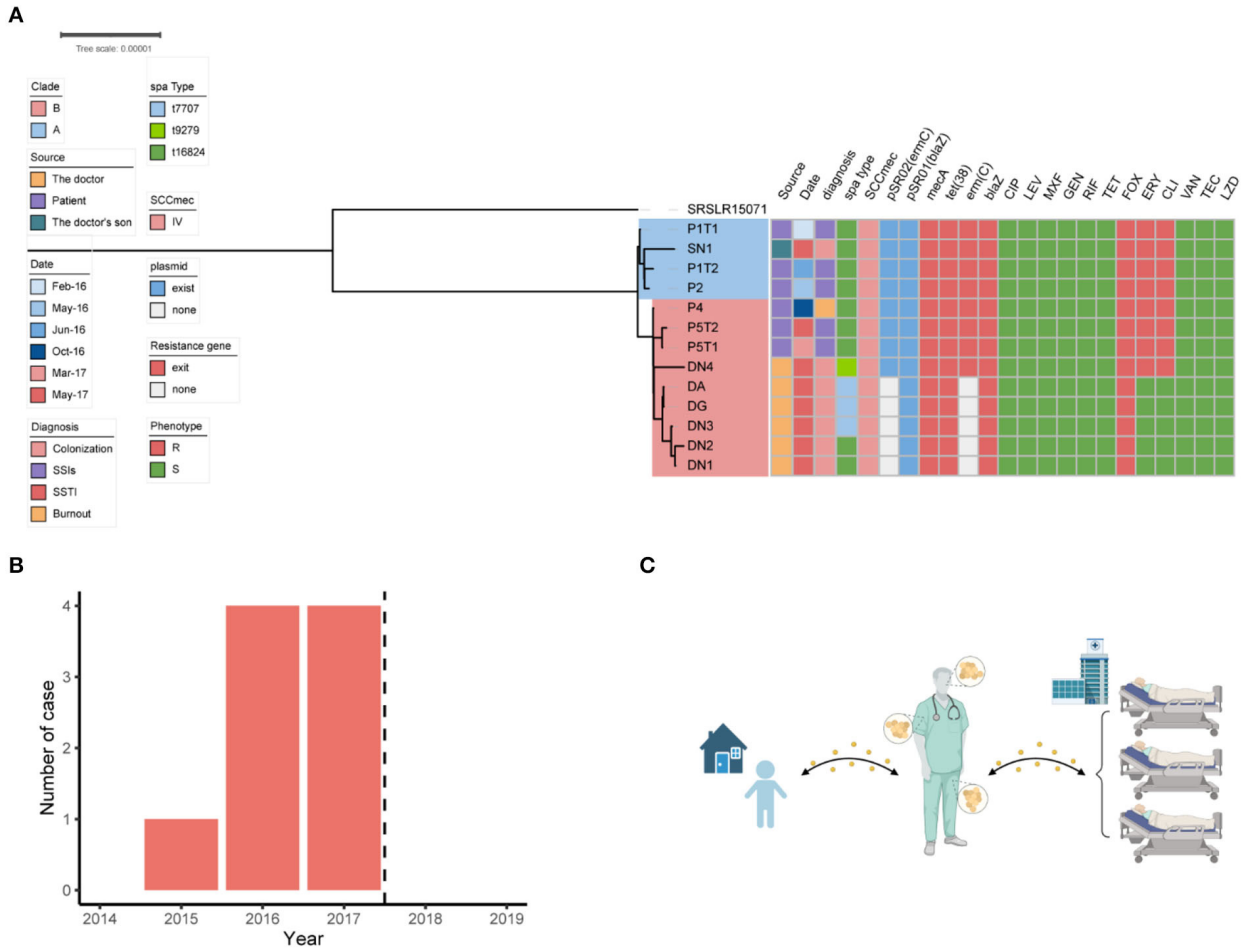
Dissemination analysis of the ST88 strain in this disease area. **(A)** Phylogenetic tree of the ST88-methicillin-resistant *Staphylococcus aureus* (MRSA) isolates disseminated in this ward. **(B)** Annual number of MRSA-infected patients in the ward. Dotted lines represent the dates when surgeons colonized with MRSA ceased their medical activity. **(C)** Transmission pattern of ST88-MRSA strains in this study.

To investigate the relationship among the ST88-MRSA isolates detected in this ward, phylogenetic analysis of the genomes of these strains was performed. The results showed that MRSA isolates from patients treated before (P1 and P2) and after (P4 and P5) October 2016 were divided into two clades: A and B. The MRSA isolates from SN1 belonged to clade A. Isolates DA, DN1, DN2, DN3, DN4, and DG, which were obtained from the different body parts of Doctor D, belonged to clade B (Figure 2A). Nine different SNVs were detected between SN1 and P1, whereas eight SNVs were detected between SN1 and P2. Single-nucleotide polymorphism analysis revealed a maximum of 21 (<40) nucleotide position differences between the ST88 isolates (Supplementary Figure 2). Using genomic information, we obtained strong evidence of the spread of these strains among patients, doctor D, and his son. Besides, transmission of these isolates between doctor D and his son may occur before P4 admission.

### 3.4. Antimicrobial susceptibility and genotype of ST88-MRSA isolates

The antimicrobial susceptibility test results showed that the ST88-MRSA isolates from patients and the son of doctor D were resistant to erythromycin, clindamycin, and methicillin. Interestingly, both erythromycin-susceptible and erythromycin-resistant ST88-MRSA isolates were from Doctor D (Figure 2A).

**Figure 3:**
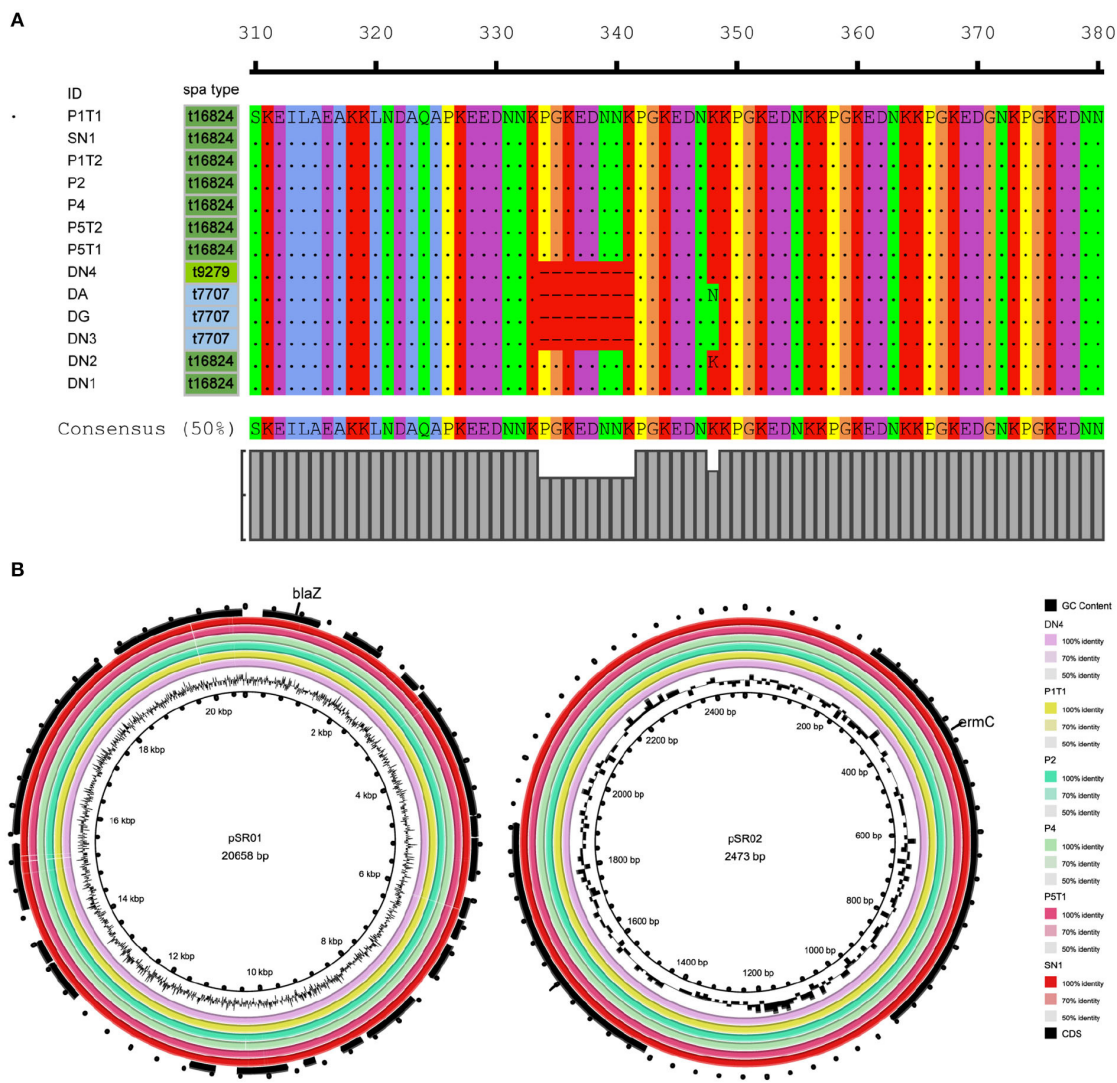
Molecular characteristics of ST88-methicillin-resistant *Staphylococcus aureus* (MRSA) isolates detected in this ward. **(A)** Amino acid sequence differences of protein A encoded by *spa*. **(B)** ST88-MRSA isolate plasmid features.

The resistance genes of the ST88-MRSA isolates were also analyzed. Staphylococcal cassette chromosome *mec* IV was carried on the chromosomes in these isolates. *ErmC* and *bla*Z were carried on two plasmids (Figure 2A). *BlaZ* was carried on the 20,658-bp plasmid pSR01, whereas *ermC* was carried on the 2,473-bp plasmid pSR02 (Figure 3B). It is noteworthy that *ermC-*carrying plasmids were absent from several isolates from Doctor D (Figure 2A).

To analyze the stability of pSR02, the ST88-MRSA strains were evaluated in a passaging experiment. After subculture in tryptic soy broth without antibiotic pressure, a clone susceptible to erythromycin was isolated from the progeny of strain P1T1 on day 11. For the DN4 and SN1 isolates, erythromycin-susceptible clones were isolated after 8 days of subculture in tryptic soy broth without antibiotic pressure. PCR analysis confirmed that the *ermC*-carrying plasmids were lost from these clones (Supplementary Table 1).

### 3.5. In-host evolution of ST88-MRSA isolates

To further analyze the in-host evolution of ST88-MRSA isolates, the molecular characteristics of these isolates were analyzed. The MRSA strains isolated from the nasal cavity of Doctor D had three different *spa* types, t16824, t9279, and t7707 (Figure 2A), whereas the patient's strains were all carrying the same *spa* type, t16824. Further analysis based on whole-genome sequencing data confirmed the presence of multiple mutations in the *spa* genes that encode protein A, including the deletion of amino acid sites 334–341 of protein A (t16824–t9279) and an N348K mutation of protein A (t16824–t7707; Figure 3A). This means that these strains may have colonized the doctor's nasal cavity for a long time, having evolved under the pressure of long-term host immunity.

In addition to the *spa* mutations, other gene mutations among the ST88 isolates were analyzed using the P1T1 strain as a reference. We detected mutations in genes related to the synthesis and structure of the *S. aureus* cell wall, colonization ability, metabolic ability, and virulence. For strains P1T1 and P1T2, which were isolated from the same patients within 4 months, mutations were identified in *fmt* and *stp* which were related to cell wall synthesis. Similarly, most acquired mutation sites in the isolates from SN1 were in genes involved in pathways related to bacterial proliferation and metabolism, such as *mgtE, polC, trpD2*, and *accB*. For strains exposed to the hospital environment for a long time, the acquired mutation sites were mainly in cell wall synthesis genes and colonization ability-related genes, such as *stp, tarK, femA, murB, sdrD*, and *essG*. For P5T1 and P5T2, which were isolated from the most recently infected patient (P5), a mutation was detected in the virulence-related gene *srtB* (Table 3).

**Table 3:** Specific amino acid difference sites of ST88-MRSA isolates in this study.

**ID**	**Date**	***fmt***	***stp***	***tarK***	***femA***	***murB***	***sdrD***	***essG***	***uvrB***	***mgtE***	***polC***	***trpD2***	***accB***	***srtB***	***fdhD***	***degA***
		**Cell wall synthesis and structure related** **(****21****–****25****)**					**Colonization-related** **(****26**, **27****)**		**Metabolic proliferation related** **(****28****–****32****)**					**Virulence-related** **(****33****)**	**Others**	
P1T1*	2016/2/25															
P1T2	2016/6/14	A85T	G100S													
P2	2016/5/16	A85T														
P4	2016/10/19				V378A			S8R								T225P
P5T1	2017/3/24				V378A			S8R						K239N	V198A	T225P
P5T2	2017/5/10			T406M	V378A			S8R						K239N	V198A	T225P
SN1	2017/5/17	A85T					D920Y			L85V	S1126R	K198N	A54V			
DA	2017/5/12				V378A			S8R								T225P
DG	2017/5/12				V378A			S8R								T225P
DN1	2017/5/11				V378A			S8R								T225P
DN2	2017/5/11				V378A			S8R								T225P
DN3	2017/5/11				V378A			S8R								T225P
DN4	2017/5/11				V378A	T272Ile		S8R	D852N							T225P

### 3.6. Intervetions to interrupt MRSA spreading

After identifying the MRSA isolates on the doctor's body, the doctor's medical activities were suspended, and decolonization was performed by rubbing disinfectant on the body surfaces. From the end of data collection (December 31, 2019), no additional cases of MRSA infection were detected in this department in the next 2 years (Figure 2B).

## 4. Discussion

*Staphylococcus aureus* is the most common pathogenic organism in infections, accounting for 26% of prosthetic joint infections and 42% of all bone and joint infections according to a previous study (34). Because of the increasing presence of antibiotic-resistant pathogens, there are no reliable treatment regimens for infections occurring after orthopedic joint surgery (35). Although vancomycin remains the first-line therapy for SSI caused by MRSA, the failure rate is as high as 35%−46% (36). Vonberg et al. (37) reported that among 191 outbreaks of *S. aureus* infection, there was a strong epidemiological evidence that *S. aureus*-colonized healthcare workers were the source in 14 outbreaks. Therefore, efforts to prevent postoperative infections in patients with joint injuries must be increased. However, the transmission mode of MRSA bone and joint infections in healthcare setting has not been widely evaluated, hindering the prevention and control of MRSA infections.

In this study, we investigated MRSA infection outbreaks in a trauma and orthopedic department. For three MRSA-induced SSI patients, although antimicrobial susceptibility tests showed that these MRSA isolates were susceptible to vancomycin and linezolid, the therapeutic effect was not satisfactory. The successive isolation of MRSA from the surgical sites of these three patients led us to suspect that there is an MRSA infection outbreak in this ward. Further investigation by MRSA screening showed that the environmental samples and hospitalized patients were negative for MRSA, but the surgeon in charge (doctor D) and his son were colonized by MRSA. Thus, we presumed that MRSA isolates could be transmitted between wards and the community *via* medical staff and their households. Using next-generation sequencing, we confirmed that MRSA isolated from patients with SSIs belonged to the ST88 clone, similar to the MRSA isolates from doctor D (and his son) who was in charge of these patients.

ST88-MRSA was previously reported as a prevalent CA-MRSA clone in Africa (38). While in Asia, previous studies showed that ST88-MRSA-IV was also the most colonized MRSA isolate from healthcare workers in Tehran, Iran (39). Furthermore, ST88-MRSA strains isolated from pigs and humans who come into contact with pigs have been reported (40). These studies suggest that ST88-MRSA is a widely distributed clone in animal husbandry and communities, with a strong colonization capacity in humans. Based on our genomic data, ST88-MRSA isolates causing nosocomial outbreaks might originated from the households of doctors. In a previous study, an SNV count of ≤ 40 was considered to indicate the same isolate (41). According to the thresholds previously reported, the ST88-MRSA isolates circulated among patients P1, P2, P4, P5, Doctor D, and Doctor D's household were closely related.

Further phylogenetic analysis showed that the ST88 isolates from the patients were divided into two clades that coincided with the timing of their acquisition. Interestingly, the MRSA isolates from SN1 had a similar genome as those detected in the early hospitalized patients (P1 and P2). This result indicates that these strains might have been transmitted between doctor D and SN1 before October 2016, which was before P4 was hospitalized. Because of the low selective pressure in the community environment, the colonized strain from SN1 evolved slowly. In contrast, the MRSA strains isolated from doctor D, which were exposed to the hospital environment, acquired more mutations than the strains from SN1. Based on these data, we presumed several possible propagation modes. First, the doctor's son spread the MRSA isolates to the doctor who transported them to the hospital, causing nosocomial transmission. Second, a patient brought the strain to the hospital and transmitted it to doctor D who brought it home and spread it to his son. At the same time, strains colonizing the nasal cavity of doctor D caused nosocomial MRSA dissemination. Third, mutual transmission between patients in a hospital is also a possible mode of transmission (Figure 2C).

Because limited MRSA strains preserved from this ward before the outbreak, it was difficult to determine the precise pattern of ST88-MRSA spread in the hospital among healthcare workers and their families. However, after the Doctor D stopping his medical activities, there were no additional cases of MRSA infection in this ward in the next 2 years. This evidenced that doctors and their households may act as reservoirs for the spread of MRSA in hospitals.

Molecular characterization of these strains supported that they have been colonizing Doctor D for a long time. The doctor's nasal cavity was colonized by three *spa* types of MRSA. As *spa* encodes protein A, which is associated with evasion of host immunity (42), the ST88-MRSA isolates may have evolved different *spa* types by interacting with the host immune system in the doctor's nasal cavity. Furthermore, monoclonal *spa* typing of isolates from the patient, the doctor's son, and other parts of the doctor was performed, but only *spa* type t16824 was detected (data not shown). Protein A encoded by *spa* is also associated with MRSA colonization, and *spa*-knockout strains showed increased IgG responses against staphylococcal colonization determinants (43). As amino acid changes in protein A may affect the colonization capacity of MRSA strains, our results revealed the evolutionary processes of the strain within a healthcare-associated worker. Moreover, MRSA strains with and without an *ermC-*carrying plasmid were present in the nasal cavity of Doctor D, and we confirmed that ST88 MRSA could lose their *ermC-*carrying plasmids in the absence of pressure, suggesting that gene loss during colonization contributed to heterogeneity in antimicrobial resistance among these closely related MRSA strains.

Further functional analysis of SNVs suggested that the evolution of ST88 strains is influenced by environmental selection. Most ST88-MRSA isolates in our study had mutations in cell wall synthesis and structure-related proteins, which may be attributed to the routine use of β-lactams to prevent infection before bone and joint surgeries in this hospital (data not shown). MRSA isolates from the doctor and his son acquired the mutation in colonization-related genes. MRSA isolated from the last patient carried mutations in the virulence-related gene *srtB*. Previous studies confirmed that *srtB* encoding Sortase B plays an important role in *S. aureus* infections (33). The backbone amide of Glu224 and the side chain of Arg233 of Sortase B are essential for enzymatic catalysis activity (44). In our isolates, the mutation Lys239Asn in Sortase B may have affected the virulence of the isolates by influencing the activity of Sortase B. Further studies are needed to determine the biological and clinical roles of these specific mutation sites.

After stopping doctor D's medical activities, there were no cases of MRSA infection in this ward during the study period. This result further validates that healthcare staff play an important role in MRSA outbreaks in healthcare settings and that expanding the screening and decolonization to the households of medical staff is also important.

There are some limitations in our research. Because of the small sample size, the origin of ST88 MRSA isolates was difficult to trace before the outbreak. Using whole gene sequencing, we obtained evidence of MRSA transmission between communities and hospitals, but the phenotypes related to the mutations detected in these ST88 MRSA are not fully known. However, our data provide valuable evidence for the invasion of CA-MRSA in healthcare settings.

## 5. Conclusion

We found that community-associated clones could invade the hospital *via* household transmission. Next-generation sequencing technology is a useful tool for tracing MRSA strains causing severe outbreaks. Furthermore, interventions such as MRSA screening and decolonization should not be limited to healthcare settings but should also be implemented in the household environment.

## Data availability statement

The datasets presented in this study can be found in online repositories. The names of the repository/repositories and accession number(s) can be found at: BioProject, PRJNA893823.

## Ethics statement

The studies involving human participants were reviewed and approved by the local Ethics Committee of Sir Run Run Shaw Hospital reviewed and approved this study (number 20190821-9). The patients/participants provided their consent to participate in this study. The patients/participants provided their written informed consent to participate in this study. Written informed consent was obtained from the individual(s), and minor(s)' legal guardian/next of kin, for the publication of any potentially identifiable images or data included in this article.

## Author contributions

YaC, YYu, and LoS designed this study. LoS, HZ, YYi, and LD collected the isolates and clinical data. HZ, XL, and ZW performed bioinformatics analysis. LoS, HZ, LD, XL, YYi, ZW, MC, SJia, YiC, FZ, and HW performed experiments. SJi, LuS, DW, YYu, and YaC supervised and directed this project. LoS, HZ, and YaC wrote the manuscript. All authors commented on the manuscript.
