# Supplementary material for: Transmission and microevolution of methicillin-resistant Staphylococcus aureus ST88 strain among patients, healthcare workers, and household contacts at a trauma and orthopedic ward
Source: Front Public Health. 2023 Jan 9;10:1053785. doi: 10.3389/fpubh.2022.1053785 (PMC9868773; doi:10.3389/fpubh.2022.1053785)
Supplement: Supplementary file 1 [file Data_Sheet_1.docx]

Supplementary Material


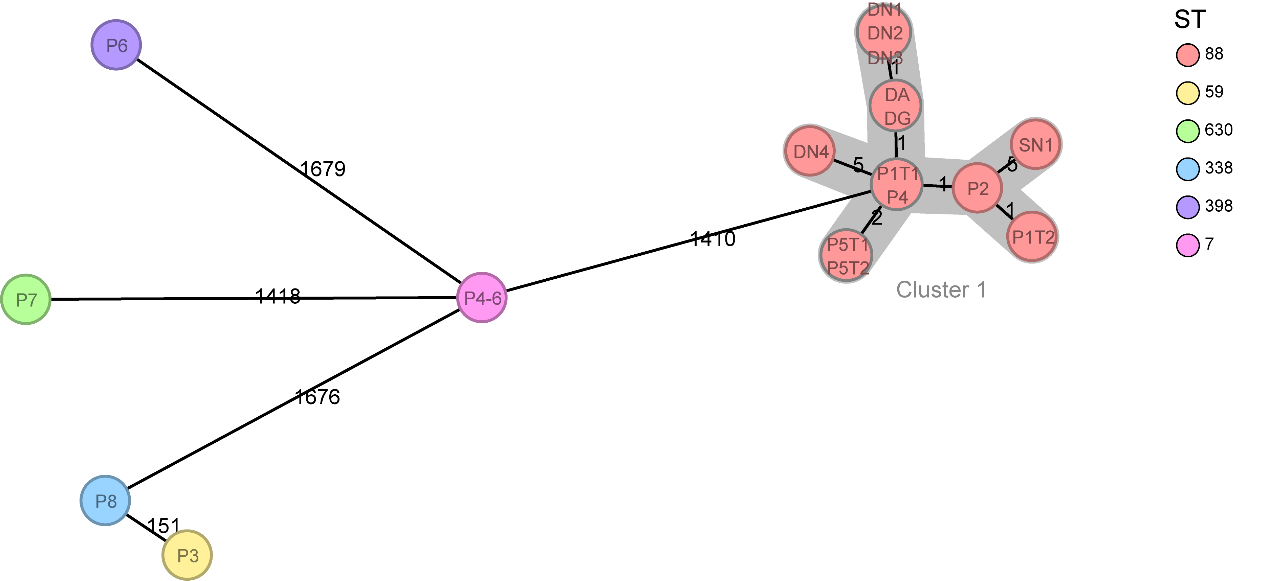


**Supplementary Figure 1.** Minimal occurrence tree of the methicillin-resistant *Staphylococcus aureus* (MRSA) strains isolated from this ward.


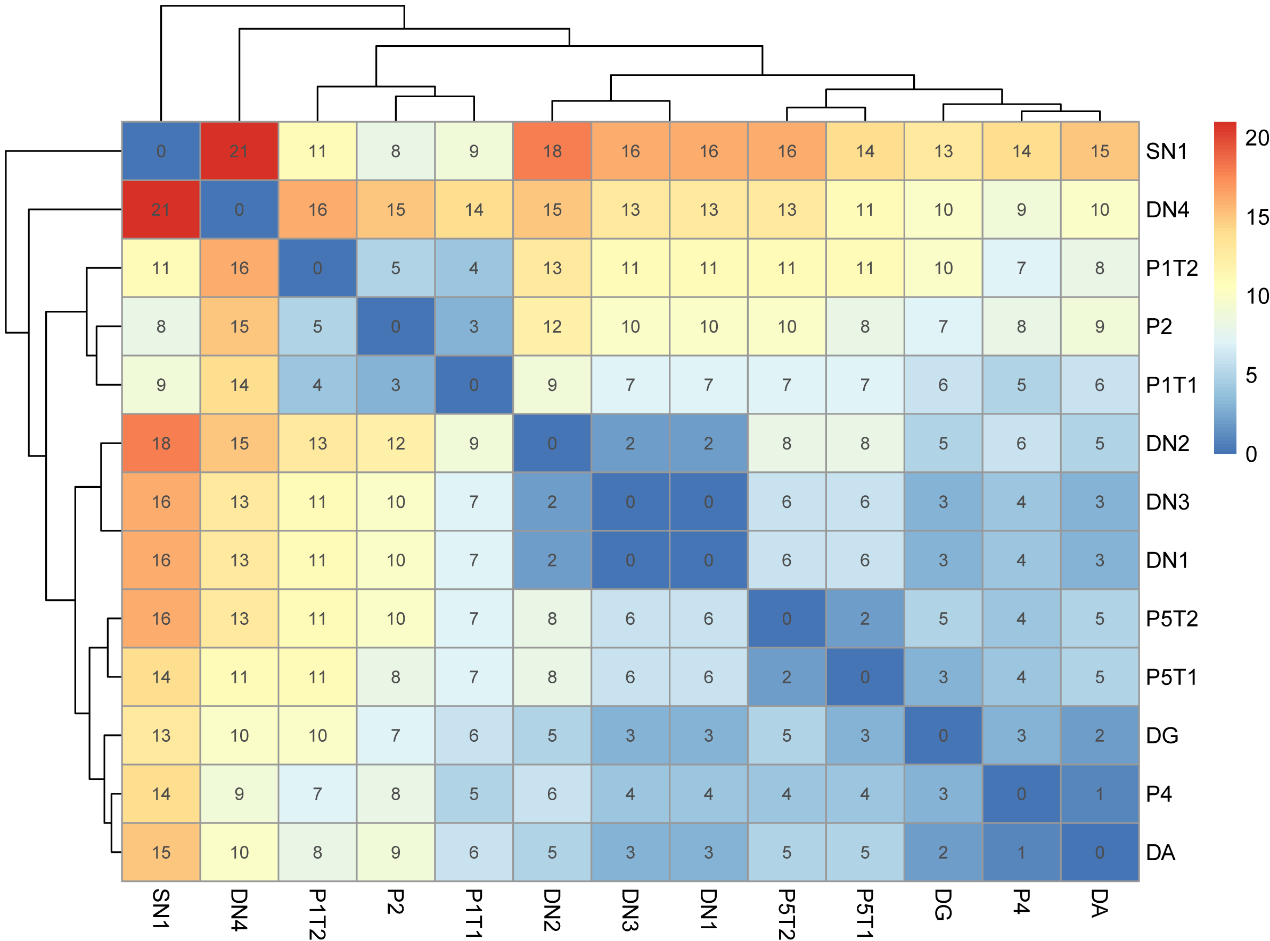


**Supplementary Figure 2.** Number of single-nucleotide polymorphism (SNP) differences in the ST88-methicillin-resistant *Staphylococcus aureus* (MRSA) strains isolated from this ward.

**Supplementary Table 1**. Antibiotic-free stress passage test of ST88-MRSA isolates

| ID of parent strain | Day on which erythromycin-susceptible colony was isolated |
| --- | --- |
| P1T1 | Day 11 |
| DN4 | Day 8 |
| SN1 | Day 8 |
